# Supplementary material for: Tau Filaments from Amyotrophic Lateral Sclerosis/Parkinsonism-Dementia Complex (ALS/PDC) adopt the CTE Fold
Source: bioRxiv. 2023 Apr 28:2023.04.26.538417. Preprint. [Version 1] doi: 10.1101/2023.04.26.538417 (PMC10168338; doi:10.1101/2023.04.26.538417)
Supplement: Supplement 1 [file NIHPP2023.04.26.538417v1-supplement-1.pdf]

bioRxiv preprint doi: https://doi.org/10.1101/2023.04.26.538417; this version posted April 28, 2023. The copyright holder for this preprint (which was not certified by peer review) is the author/funder, who has granted bioRxiv a license to display the preprint in perpetuity. It is made available under aCC-BY 4.0 International license.

SUPPLEMENTARY FIGURES

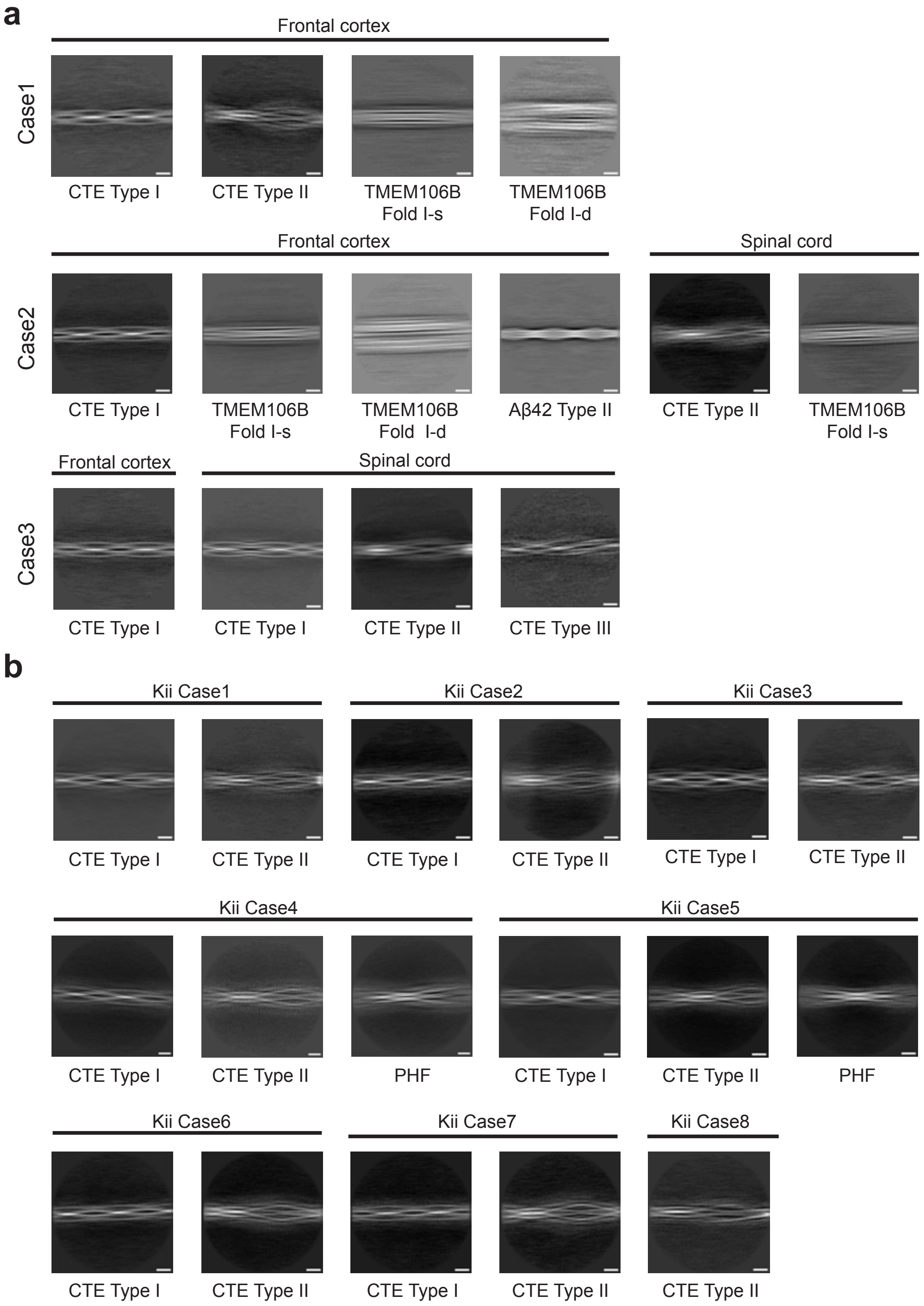

Supplementary Figure 1: Two-dimensional classification of filaments from Guam (a) and Kii (b) ALS/PDC.

Scale bar, 10 nm.

bioRxiv preprint doi: https://doi.org/10.1101/2023.04.26.538417; this version posted April 28, 2023. The copyright holder for this preprint (which was not certified by peer review) is the author/funder, who has granted bioRxiv a license to display the preprint in perpetuity. It is made available under aCC-BY 4.0 International license.

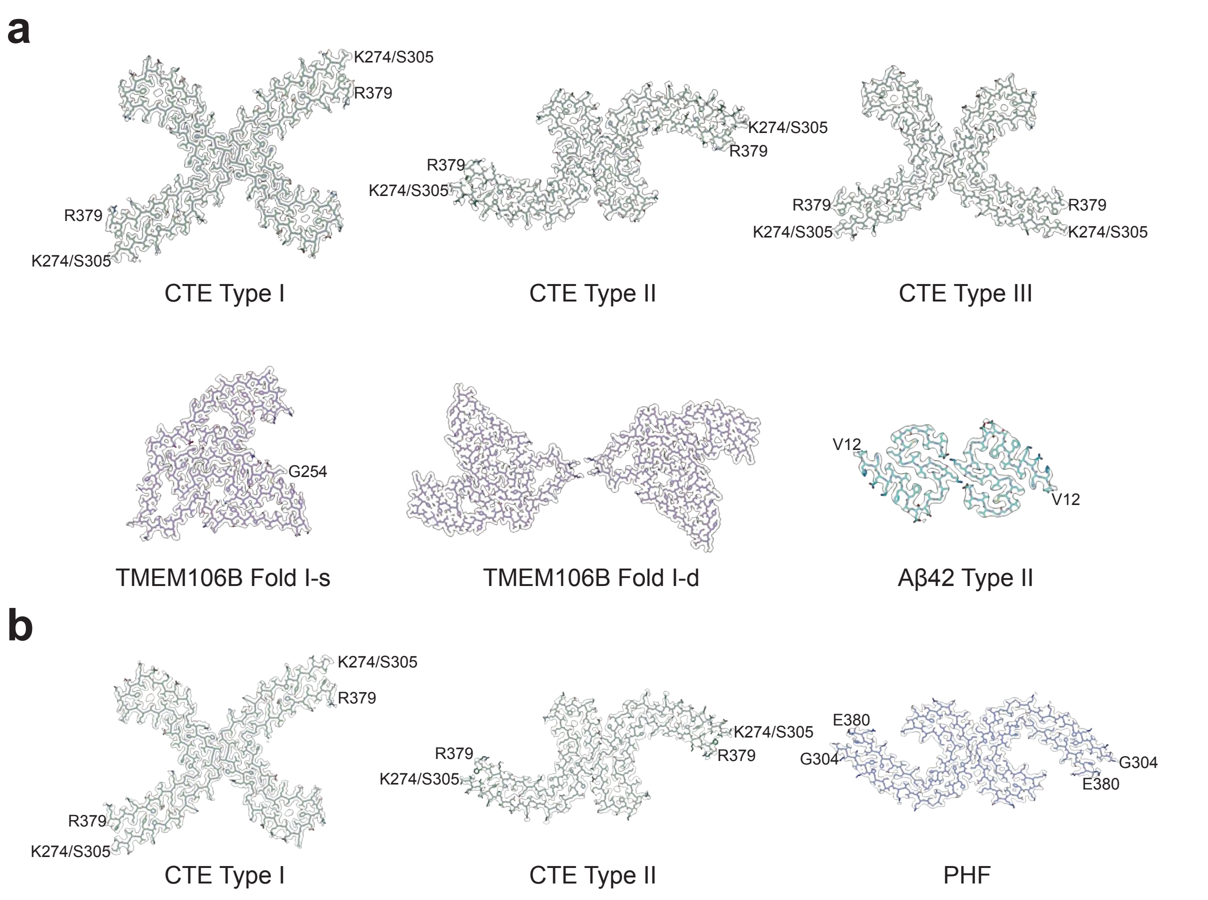

Supplementary Figure 2: Cryo-EM density maps of filaments from Guam and Kii ALS/PDC.

(a), In Guam ALS/PDC, Type I, Type II and Type III tau filaments (green), singlets and doublets of TMEM106B filaments (fold I) (purple) and Type II Aβ42 filaments (cyan) were present.
(b), In Kii ALS/PDC, Type I and Type II tau filaments (green), as well as tau paired helical filaments (PHFs) (blue), were present.

bioRxiv preprint doi: https://doi.org/10.1101/2023.04.26.538417; this version posted April 28, 2023. The copyright holder for this preprint (which was not certified by peer review) is the author/funder, who has granted bioRxiv a license to display the preprint in perpetuity. It is made available under aCC-BY 4.0 International license.

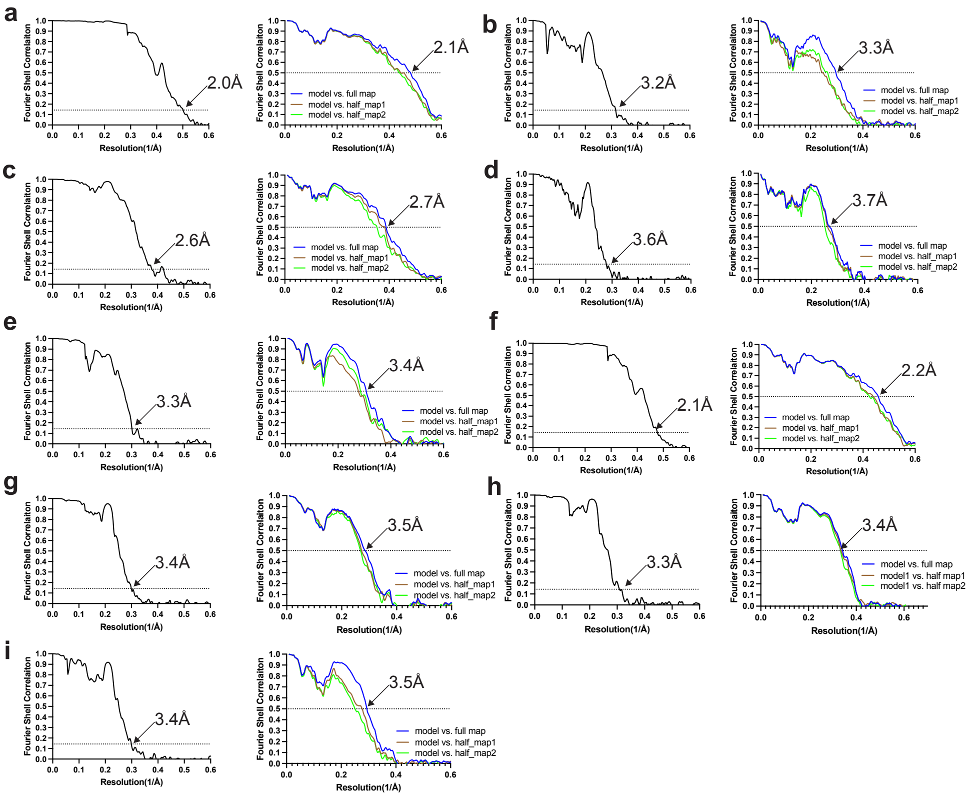

Supplementary Figure 3: Fourier shell correlation (FSC) curves.

FSC curves of cryo-EM maps (left panel) and model to map validation (right panel). **(a)**, Guam ALS/PDC CTE tau Type I. **(b)**, Guam ALS/PDC CTE tau Type II. **(c)**, Guam ALS/PDC TMEM106B fold I-s. **(d)**, Guam ALS/PDC TMEM106B fold I-d. **(e)**, Guam ALS/PDC CTE tau Type II Aβ42. **(f)**, Kii ALS/PDC CTE tau Type I. **(g)**, Kii ALS/PDC CTE tau Type II. **(h)**, Kii ALS/PDC tau PHF. **(i)**, Guam ALS/PDC CTE tau Type III.

bioRxiv preprint doi: https://doi.org/10.1101/2023.04.26.538417; this version posted April 28, 2023. The copyright holder for this preprint (which was not certified by peer review) is the author/funder, who has granted bioRxiv a license to display the preprint in perpetuity. It is made available under aCC-BY 4.0 International license.

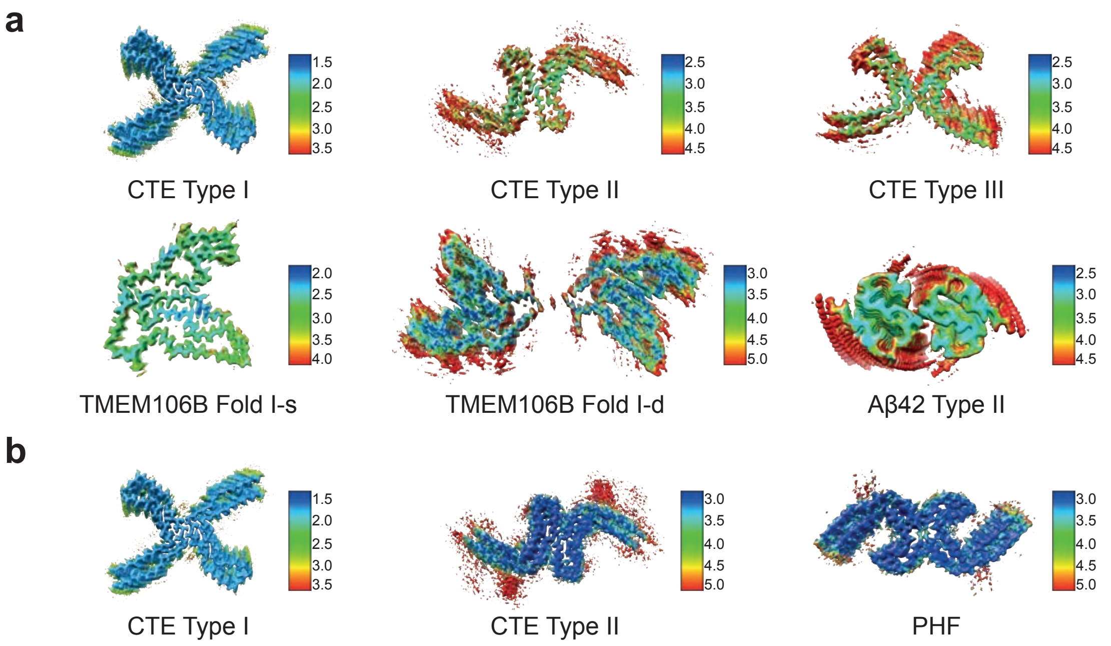

Supplementary Figure 4: Local resolution estimation of filaments from Guam (a) and Kii (b) ALS/PDC.

bioRxiv preprint doi: https://doi.org/10.1101/2023.04.26.538417; this version posted April 28, 2023. The copyright holder for this preprint (which was not certified by peer review) is the author/funder, who has granted bioRxiv a license to display the preprint in perpetuity. It is made available under aCC-BY 4.0 International license.

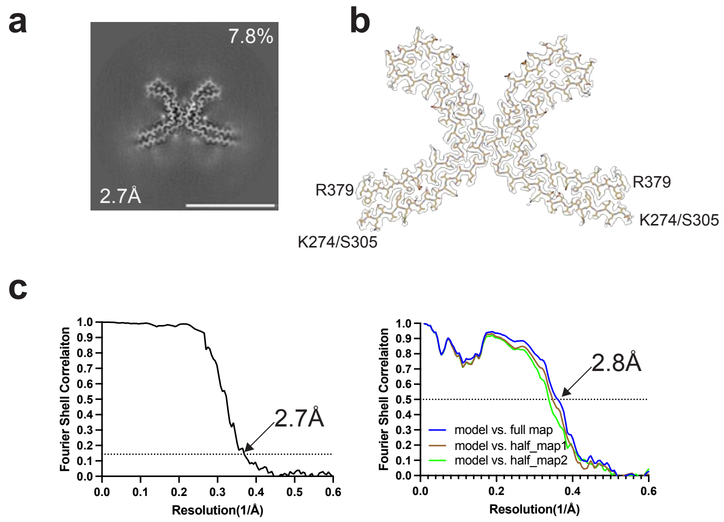

Supplementary Figure 5: Type III filaments from CTE.

(a), Cross-section perpendicular to the helical axis of the cryo-EM structure of Type III filaments from temporal cortex of CTE case 2 (30), with a projected thickness of approximately one rung along the helical axis. **(b)**, Cryo-EM density map and model of Type III filament. **(c)**, Fourier shell correlation curves of cryo-EM maps of Type III filaments (left panel) and model to map validation (right panel).

bioRxiv preprint doi: https://doi.org/10.1101/2023.04.26.538417; this version posted April 28, 2023. The copyright holder for this preprint (which was not certified by peer review) is the author/funder, who has granted bioRxiv a license to display the preprint in perpetuity. It is made available under aCC-BY 4.0 International license.

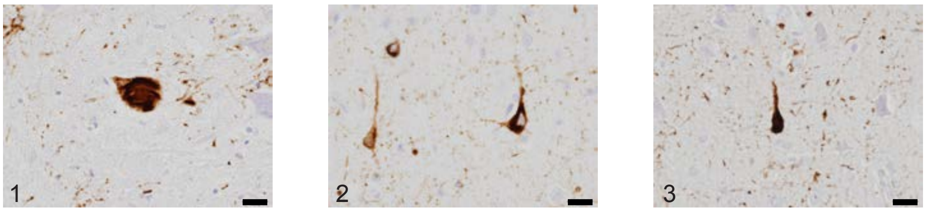

Supplementary Figure 6: Immunostaining of tau inclusions from Guam ALS/PDC.

Sections from the frontal cortex of cases 1-3 stained with anti-tau antibody AT8. Scale bar, 20 μm.

bioRxiv preprint doi: https://doi.org/10.1101/2023.04.26.538417; this version posted April 28, 2023. The copyright holder for this preprint (which was not certified by peer review) is the author/funder, who has granted bioRxiv a license to display the preprint in perpetuity. It is made available under aCC-BY 4.0 International license.

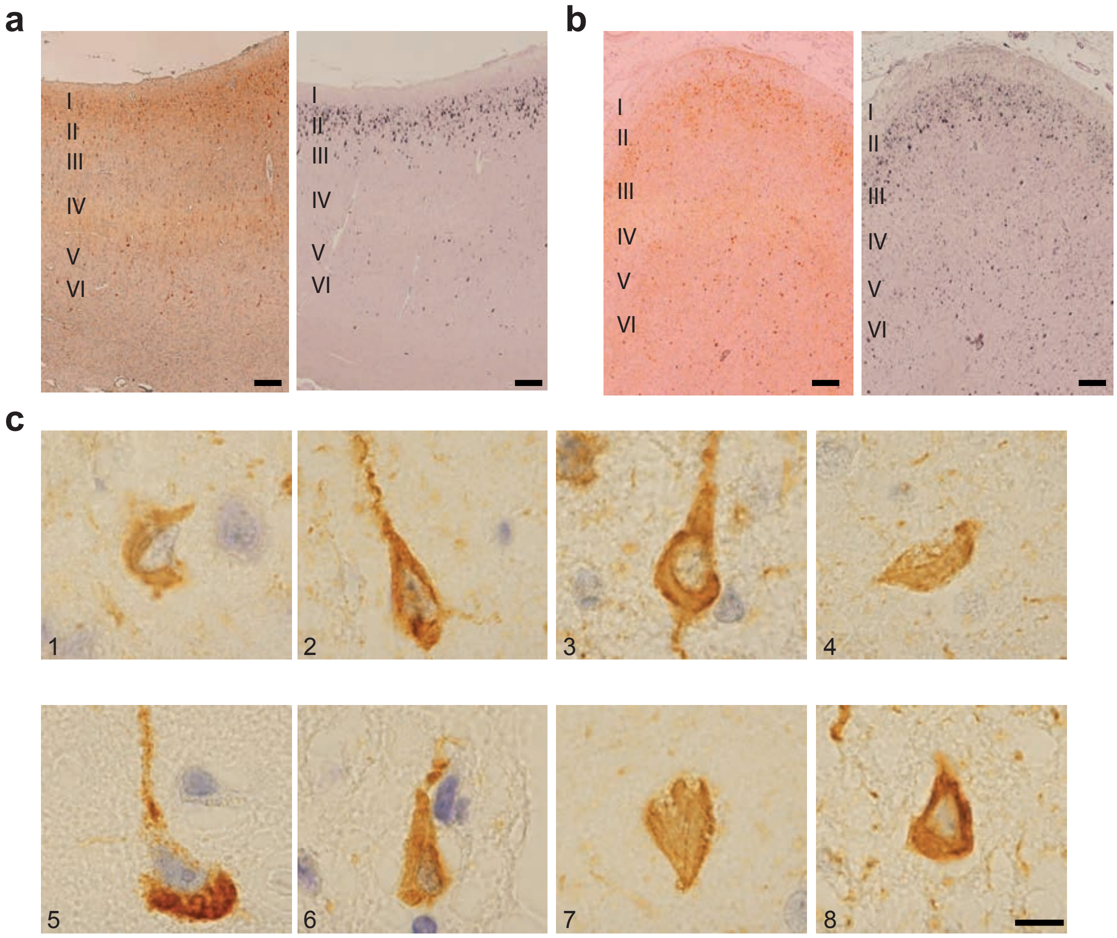

Supplementary Figure 7: Immunostaining of tau inclusions from Kii ALS/PDC.

(a,b), Temporal cortex from cases 3 and 4 stained with anti-tau antibody AT8 (left) and with Gallyas-Braak silver (right). Tau inclusions are concentrated in layers II/III. Scale bar, 200 μm.
(c), Sections from the temporal cortex of cases 1-8 stained with AT8. Scale bar, 10 μm.

bioRxiv preprint doi: https://doi.org/10.1101/2023.04.26.538417; this version posted April 28, 2023. The copyright holder for this preprint (which was not certified by peer review) is the author/funder, who has granted bioRxiv a license to display the preprint in perpetuity. It is made available under aCC-BY 4.0 International license.

SUPPLEMENTARY TABLES

Supplementary Table 1: Cryo-EM data collection, refinement and validation statistics for Guam ALS/PDC.

	Guam Case3 spinal cord		Guam Case1 frontal cortex		Guam Case2 frontal cortex	
**Data collection**						
Microscope	Titan Krios		Titan Krios		Titan Krios	
Voltage (kV)	300		300		300	
Detector	Falcon4		Falcon4		Falcon4	
Magnification	96,000		96,000		96,000	
Electron exposure (e–/Å^2)	40		40		40	
Defocus range (μm)	-1.0 to -2.0		-1.0 to -2.0		-1.0 to -2.0	
Pixel size (Å)	0.824		0.824		0.824	
**Data processing**	CTE TypeI	CTE TypeIII	CTE type II	TMEM106B Fold I-s	TMEM106B Fold I-d	Aβ42 TypeII
Box size (pixel)	400	400	400	400	400	400
Symmetry imposed	C1	C1	C1	C1	C1	C2
Initial particle images (no.)	256,999		130,240		281,921	
Final particle images (no.)	140,124	13,456	3,105	57,802	11,131	20,363
Map resolution (Å)<br>FSC threshold 0.143	2.0	3.4	3.2	2.6	3.6	3.3
Helical rise (Å)	2.37	4.74	2.38	4.81	4.79	4.79
Helical twist (°)	179.41	-1.19	179.4	-0.4	-0.42	-2.99
**Refinement**						
Model resolution (Å)<br>FSC threshold 0.5	2.1	3.5	3.3	2.7	3.7	3.4
Map sharpening *B* factor (Å^2)	-26	-54	-30	-28	-69	-43
Model composition<br>Non-hydrogen atoms<br>Protein residues<br>Ligands	 2870<br>375<br>0	 3444<br>450<br>0	 3444<br>450<br>0	 4340<br>540<br>0	 8680<br>1080<br>0	 1356<br>186<br>0
B factors (Å^2)<br>Protein	 104.2	 196.3	 312.4	 141.1	 222.2	 156.8
R.m.s. deviations<br>Bond lengths (Å)<br>Bond angles (°)	 0.004<br>1.006	 0.007<br>1.541	 0.0056<br>1.11	 0.0056<br>1.367	 0.0071<br>1.537	 0.0073<br>1.369
Validation<br>MolProbity score<br>Clashscore<br>Poor rotamers (%)	 0.95<br>0.51<br>1.52	 2.06<br>3.56<br>6.82	 1.39<br>1.99<br>1.52	 2.21<br>4.15<br>4.76	 2.54<br>5.19<br>5.95	 2.17<br>4.71<br>4.35
Ramachandran plot<br>Favored (%)<br>Allowed (%)<br>Disallowed (%)	 97.26<br>2.74<br>0	 95.89<br>4.11<br>0	 95.89<br>4.11<br>0	 91.73<br>8.27<br>0	 84.21<br>15.79<br>0	 93.1<br>6.9<br>0
PDB	8OT6	8OT9	8OTC	8OTD	8OTE	8OTF
EMDB	EMD-17171	EMD-17173	EMD-17174	EMD-17175	EMD-17176	EMD-17177

bioRxiv preprint doi: https://doi.org/10.1101/2023.04.26.538417; this version posted April 28, 2023. The copyright holder for this preprint (which was not certified by peer review) is the author/funder, who has granted bioRxiv a license to display the preprint in perpetuity. It is made available under aCC-BY 4.0 International license.

Supplementary Table 2: Cryo-EM data collection, refinement and validation statistics for Kii ALS/PDC and CTE.

	Kii Case5			CTE Case2
**Data collection**				
Microscope	Titan Krios			Titan Krios
Voltage (kV)	300			300
Energy filter slit (eV)	20			20
Detector	K3			Falcon4
Magnification	105,000			96,000
Electron exposure (e–/Å^2)	40			40
Defocus range (μm)	-1.0 to -2.0			-1.2 to -2.4
Pixel size (Å)	0.826			0.824
**Data processing**	CTE typeI	CTE typeII	PHF	CTE Type III
Box size (pixel)	400	400	400	256
Symmetry imposed	C1	C1	C1	C1
Initial particle images (no.)	414,439			538,420
Final particle images (no.)	181,144	33,337	39,424	41,955
Map resolution (Å)<br>FSC threshold 0.143	2.1	3.4	3.3	2.7
Helical rise (Å)	2.39	2.39	2.38	4.78
Helical twist (°)	179.39	179.36	179.45	-1.14
**Refinement**				
Model resolution (Å)<br>FSC threshold 0.5	2.2	3.5	3.4	2.8
Map sharpening *B* factor (Å^2)	-31	-84	-73	-55
Model composition<br>Non-hydrogen atoms<br>Protein residues<br>Ligands	 2870<br>375<br>0	 2870<br>375<br>0	 4109<br>539<br>0	 3444<br>450<br>0
B factors (Å^2)<br>Protein	 101.6	 247.6	 238.6	 199.6
R.m.s. deviations<br>Bond lengths (Å)<br>Bond angles (°)	 0.0063<br>0.91	 0.0065<br>1.378	 0.0067<br>1.593	 0.0103<br>1.517
Validation<br>MolProbity score<br>Clashscore<br>Poor rotamers (%)	 1.3<br>1.02<br>3.03	 2.15<br>8.88<br>1.52	 2.53<br>7.78<br>4.48	 1.81<br>3.27<br>5.30
Ramachandran plot<br>Favored (%)<br>Allowed (%)<br>Disallowed (%)	 97.26<br>2.74<br>0	 90.41<br>9.59<br>0	 87.62<br>12.38<br>0	 97.26<br>5.30<br>0
PDB	8OTG	8OTH	8OTJ	8OTI
EMDB	EMD-17178	EMD-17179	EMD-17181	EMD-17180
